# Polymer Optofluidic Chip for Double-Beam Photometric Determination of Nitrite in Water Samples

**DOI:** 10.1021/acsomega.6c02297

**Published:** 2026-08-05

**Authors:** Vladislav Reimer, Zhenyu Zhang, Christoff Brüdigam, Martin Angelmahr, Eike Hübner, Wolfgang Schade

**Affiliations:** † Institute of Energy Research and Physical Technologies, 26534 Clausthal University of Technology, Leibnizstrasse 4, 38640 Goslar, Germany; ‡ Fiber Optical Sensor Systems, 28482 Fraunhofer Heinrich Hertz Institute, HHI, Am Stollen 19H, 38640 Goslar, Germany; § Institute of Organic Chemistry, Clausthal University of Technology, Leibnizstrasse 6, 38678 Clausthal-Zellerfeld, Germany

## Abstract

We present a compact polymer optofluidic lab-on-a-chip fabricated by maskless laser-direct UV lithography for double-beam photometric detection of nitrite (NO_2_^–^) in water. The chip integrates polymer optical waveguides, a 3 dB coupler, and dual microfluidic cells, implementing on-chip measurement and reference channels that compensate for light-source fluctuations and improve the robustness of the photometric measurements. The system is operated with commercial Griess-reaction-based test kits and standard RGB CMOS image sensors configured for selective RGB-channel absorbance readout. The system achieves a limit of detection of 0.08 mg L^–1^ of nitrite in reagent-treated standard water samples. These results highlight the potential of low-cost polymer optofluidic chips as portable platforms for nitrite monitoring in drinking water and environmental applications.

## Graphical abstract

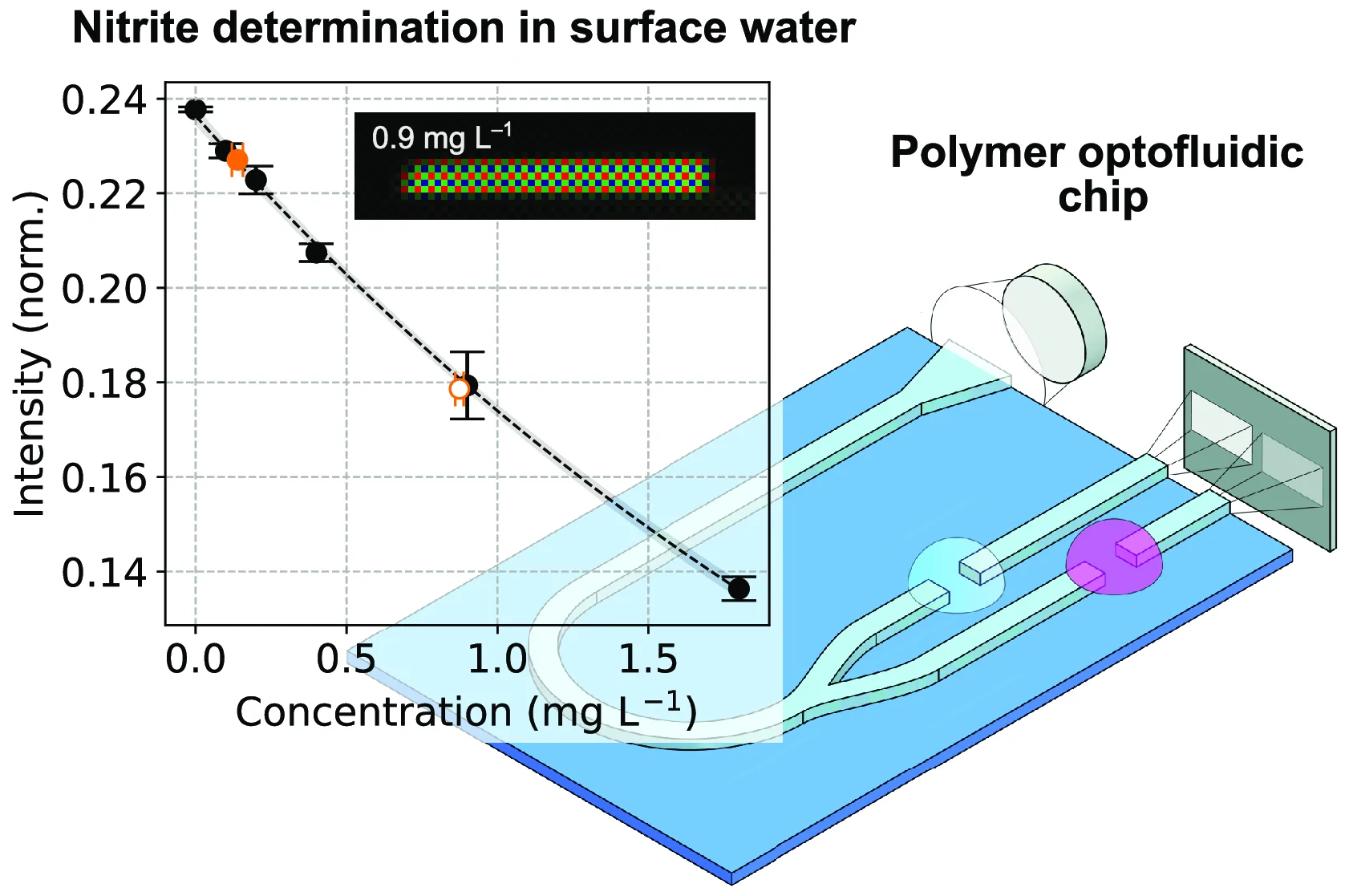

## 1 Introduction

Reliable, frequent, and spatially distributed monitoring of water quality across supply, treatment, and reuse stages is an important part of the transition from a linear to a circular water economy model.^1,2^ Cost-effective, compact systems for on-site water analysis are crucial for timely decision-making, enforcing regulatory limits, and ensuring the safe reuse of treated water, while reducing reliance on labor-intensive laboratory testing. Conventional monitoring requires sample collection and transport to centralized laboratories for preparation, measurement, and documentation, which hinders real-time field testing and consumes substantial resources. These analyses often rely on colorimetric methods that involve extensive sample handling and relatively large reagent volumes. Portable, selective on-site systems can address these limitations by reducing reagent consumption and enabling faster, more efficient water quality monitoring.^3,4^

This study focuses on monitoring nitrite (NO_2_^–^) concentrations in water, a key indicator for both drinking-water safety and the environmental performance of agricultural and wastewater systems. Nitrite ions occur naturally in surface and groundwater, but their levels can increase markedly due to agricultural activities, industrial emissions, and domestic wastewater discharges, posing risks to human health and the aquatic ecosystems.^5–7^ Consequently, accurate and selective monitoring in natural waters is essential. The European Drinking Water Directive specifies maximum admissible concentration of 0.5 mg L^–1^ for NO_2_^–^ in drinking water.^8^

A major recent advance in on-site analysis is the integration of different hand-held devices, such as commercial smartphones,^9,10^ and laboratory-demonstrated colorimeters^11^ and analyzers,^12^ to create compact measurement platforms. These devices offer onboard computation, built-in light sources, and cameras that serve as emitters and detectors in optical analysis systems, and they can transmit data to remote servers in real time. Microfluidic chips, typically centimeter-scale devices fabricated from silicon, glass, or polymers, enable controlled liquid handling and on-chip analysis and have been used for on-site nitrite determination using electrochemical and optical methods.^4^ Electrochemical methods provide high sensitivity and selectivity but often require complex fabrication of chemically modified electrodes and careful control of measurement conditions.^13–16^ In contrast, optical methods based on colorimetric reactions are simpler to implement, inherently compatible with low-cost light sources and cameras, and thus well suited for hand-held-assisted sensing.^4^

Optical nitrite determination is commonly implemented either as visual colorimetry or as photometry. In visual colorimetry, concentrations are estimated by comparing the color of test media, such as microfluidic paper-based analytical devices (μPADs), with color standards,^17–19^ or by quantifying color using image-capturing devices.^20,21^ Although μPADs are simple and inexpensive, their sensitivity is limited by human visual perception, and image-based readout often requires controlled lighting, especially at low analyte concentrations, to ensure accurate and consistent results.^22^

Photometry-based platforms extend colorimetric analysis by performing quantitative absorbance measurements: reagent-based colorimetric reactions generate species that absorb light at specific wavelengths, and the measured absorbance is proportional to analyte concentration according to the Beer–Lambert law. Several hand-held photometric approaches for nitrite determination have been reported,^11,12,23–30^ frequently using smartphones for data acquisition while employing external light sources, discrete optical components, or separate photodetectors for signal generation and readout. In many cases, a true zero-absorbance baseline is not available, and therefore intensity drifts of the light source or changes in coupling conditions can limit accuracy and calibration during on-site applications. These limitations motivate fully integrated, compact, and scalable optofluidic systems that (i) implement on-chip reference measurements for baseline correction, (ii) are compatible with hand-held hardware, and (iii) provide the analytical performance required to meet regulatory requirements for drinking-water monitoring.

Here, we demonstrate and validate a polymer optofluidic lab-on-a-chip for determination of nitrite concentrations in water samples. The chip operates passively, using a white light-emitting diode (LED) as a broadband visible light source and a complementary metal-oxide-semiconductor (CMOS) image sensor configured as a detector for selective red–green–blue (RGB) channel absorbance readout. The device is designed to be compact, cost-effective, and suitable for reproducible large-scale production. The polymer-based chip is fabricated by maskless laser-direct UV lithography and integrates an optical waveguide, a 3 dB coupler (50:50 optical power splitter), and an optical taper. The 3 dB coupler provides two identical microfluidic detection cells, as schematically illustrated in Figure 1a, realizing an on-chip double-beam photometric configuration in which two fluidic channels provide simultaneous measurement and reference paths. This enables a double-beam-like absorbance measurement that is well-known to compensate for source and detector drift, because the reference beam allows continuous correction of the source intensity, thereby improving baseline stability and measurement precision.^31^ Similar on-chip architecture with separate measurement and reference paths has previously been demonstrated, showing intrinsic noise and drift reduction, improved stability of absorbance measurements, and a lower limit of detection compared to a single-cell waveguide sensor.^32^

**Figure 1:**
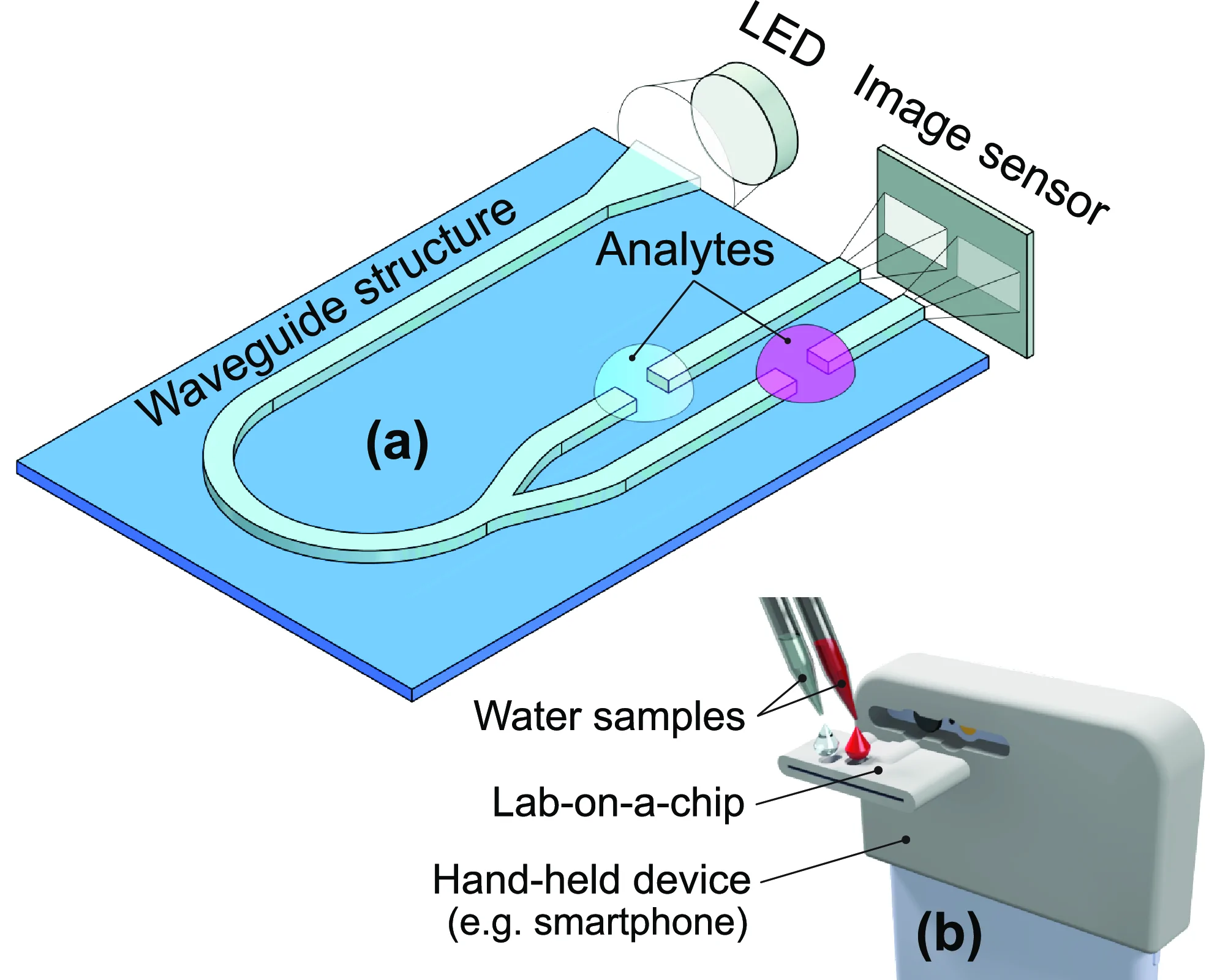
(a) Schematic of the polymer optofluidic lab-on-a-chip showing the U-shaped optical waveguide, integrated 3 dB coupler, optical taper, and two microfluidic detection cells corresponding to the measurement and reference channels. (b) Concept of a removable cartridge that positions the chip in front of the smartphone flash LED and camera for illumination and detection.

In this proof-of-concept study, we implement the optical readout using the smartphone flash LED in combination with a compact CMOS camera development board. The lab-on-a-chip is, in principle, smartphone-compatible, as shown in Figure 1b, but a fully smartphone-based implementation was beyond the scope of the present study for the reasons discussed later in this work.

## 2 Materials and Methods

### 2.1 Chip Fabrication

The polymer optofluidic chip was fabricated using a Heidelberg Instruments μPG 101 maskless lithography system. The chip comprises a 30 mm × 20 mm cyclic olefin copolymer (COC, TOPAS) substrate, (thickness 250 μm, refractive index 1.533) and an integrated L-shaped EpoCore waveguide (EpoCore/EpoClad series, Microresist GmbH). The waveguide is 500 μm wide and 50 μm high (refractive index 1.542) and incorporates an optical taper, a 3 dB coupler, and two identical 3 mm long detection cells, as shown in Figure 2a.

**Figure 2:**
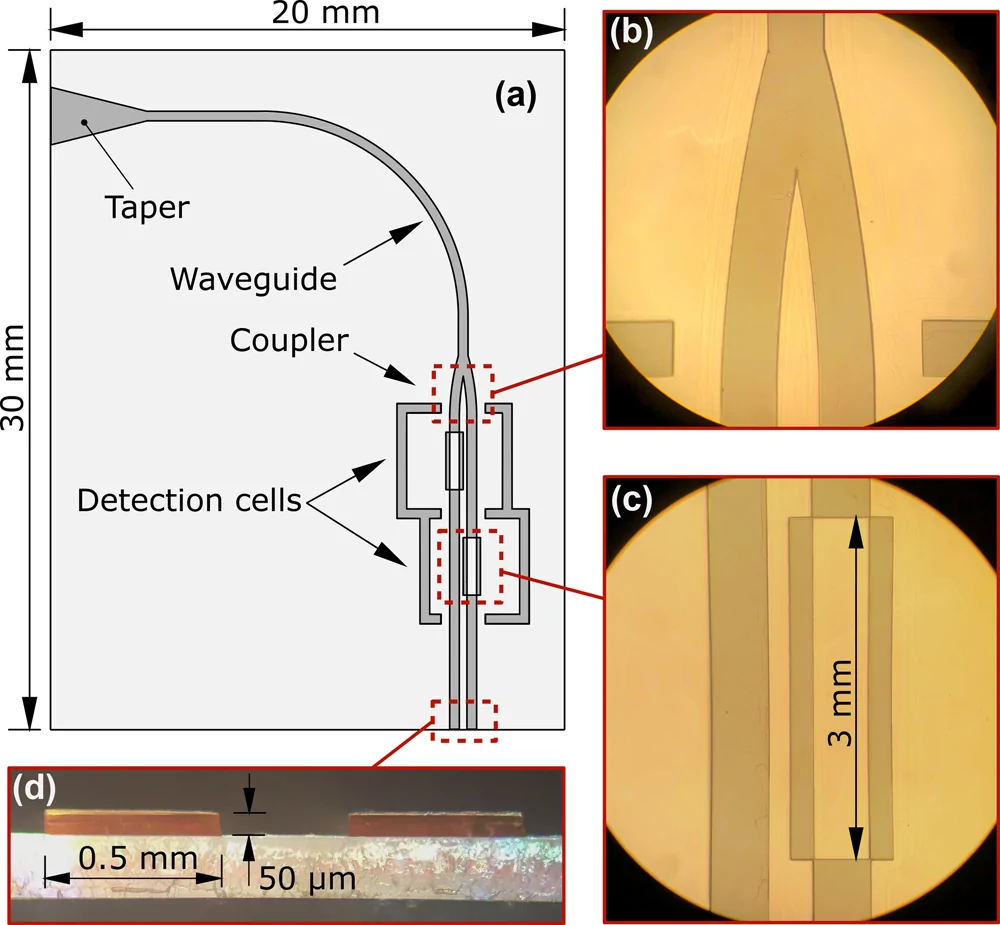
Design and microscope images of the polymer optofluidic chip. (a) Top-view schematic of the L-shaped optical waveguide, including the input taper, 3 dB coupler, and two 3 mm long detection cells. (b) Microscope image of the integrated 3 dB coupler region. (c) Microscope image of the detection cell, showing the 3 mm interruptions of the waveguide and surrounding liquid-confining frame. (d) Cross-sectional microscope image of the cleaved waveguide output facet, illustrating the 500 μm width and 50 μm height.

The optical taper improves coupling efficiency. The 3 dB coupler (Figure 2b) splits the waveguide into two paths, forming a second detection cell for reference measurements and thereby implementing a double-beam photometric configuration on chip with simultaneous measurement and reference channels for baseline correction. Each detection cell is a 3 mm long interruption of the waveguide (Figure 2c), surrounded by a frame (Figure 2a) that confines the liquid sample and prevents spreading over the chip. The waveguide input and output facets were cleaved following the procedure described in Reference.^33^ A microscope image of the cleaved output facet is shown in Figure 2d. An L-shaped waveguide was adopted instead of the U-shaped concept in Figure 1b to meet the constraints of the experimental setup. The lithography system used can reliably fabricate polymer chips up to approximately 30 mm × 30 mm. Placing both the smartphone flash LED and the CMOS camera development board on the same side, as required for a U-shaped layout, would have required a separation between the camera and LED exceeding this maximum chip length.

### 2.2 Reagents

For nitrite determination, five NO_2_^–^ standard solutions with concentrations in the range 0–10 mg L^–1^ were prepared using sodium nitrite and distilled water. The 1.14774 MQuant Nitrite Test Kit (Merck KGaA) was used to measure nitrite concentrations in the solutions. This test kit is based on the Griess reaction, wherein nitrite ions react with sulfanilic acid and an amine to form a violet azo dye. Approximately 200 μL of each treated sample and the corresponding blank was pipetted into the measurement and reference cells, respectively. After each measurement, the cells were flushed several times with fresh sample to avoid carryover. For external reference, all samples were analyzed with a DR3900 spectrophotometer (Hach Lange GmbH) to obtain NO_2_^–^ concentration values.

Experiments were performed with standard solutions in distilled water to isolate chip performance from matrix effects and with the tap and surface water to evaluate the performance of the proposed polymer-waveguide chip in representative real-water matrices. According to the reagent documentation for the 1.14774 MQuant test kits (Hach Lange GmbH; Merck KGaA),^34,35^ common inorganic ions and organic constituents at typical drinking- and environmental-water levels are not expected to cause significant interference under our conditions.

### 2.3 CMOS Sensor

The OV5640 is a low-cost CMOS image sensor with a sensitivity of 600 mV lux^–1^ s^–1^ that provides 5 megapixel, 10-bit Bayer-pattern color images and a quantum efficiency of approximately 30–50% in the visible range.^36^ The sensor supports raw Bayer output, allowing direct access to individual red, green, and blue pixel values without interpolation so that the most sensitive color channel can be selected for the measurements. Pixel intensities were recorded as 8-bit values. For each color channel *k* (red, green, blue), the mean normalized intensity was computed as

1 $$I_{k,\mathrm{norm}}=\frac{1}{N\times 256}\sum_{i=1}^{N}\left[I_{k,\mathrm{meas}}^{\left(i\right)}/I_{k,\mathrm{ref}}^{\left(i\right)}\right]$$

where *I*_*k*,norm_ is the normalized intensity of channel *k*, *I*_*k*,meas_^(*i*)^ and *I*_*k*,ref_^(*i*)^ are the 8-bit pixel values in the measurement and reference regions, respectively, and *N* is the total number of pixels used for averaging.

### 2.4 Measurement Principles

The measurement principle is based on the Beer–Lambert–Bouguer law.^37,38^ Light coupled into the waveguide is attenuated while propagating through the absorption cell due to absorption by the sample. The transmitted intensity *I*_t_ is given by

2 $$I_{t}=I_{0}e^{-\varepsilon \left(\lambda \right)\mathrm{cd}}$$

where *I*_0_ is the incident intensity, ε­(λ) is the molar absorption coefficient at wavelength λ, *c* is the analyte concentration, *d* is the optical path length. This relationship forms the basis for quantifying analyte concentrations in the sample.

For the Beer–Lambert calibration, normalized intensity ratios *I*/*I*_0_ were transformed to absorbance-like values according to

3 $$A=-\log_{10}\left(\frac{I}{I_{0}}\right)$$

where A is the absorbance-like signal, and *I* and *I*_0_ denote the RGB-channel intensities of the sample and the blank (reference), respectively. A linear model *A* = *a* + *bC* was fitted by ordinary least-squares over the calibration range, where *C* is the nitrite concentration. The slope *b* and intercept *a*, together with their standard errors and 95% confidence intervals (CI), were obtained from the linear regression.

The sensitivity was taken as the absolute value of the slope |*b*|. The limit of detection (LoD) and limit of quantification (LoQ) were estimated from the calibration curve as^39^

4 $$\mathrm{LoD}=3\frac{A_{0}}{\left|b\right|},\;\mathrm{LoQ}=10\frac{A_{0}}{\left|b\right|}$$

where *A*_0_ the standard deviation of the blank response.

## 3 Experimental Procedures

### 3.1 Photometric Chip Characteristics

Polymer waveguides are known to exhibit increased absorption below about 450 nm, which restricts their use in the UV–violet range. To ensure efficient operation with the chosen LED and nitrite test kits, we compared the EpoCore waveguide transmission spectrum and the LED emission spectrum with the UV–vis absorption band of the nitrite test solution containing the Griess-based reagent, which exhibits absorption maxima for reacted NO_2_^–^ between 525 and 560 nm.^40^ The comparison is shown in Figure 3: the black curve represents the waveguide transmittance, the blue curve the normalized LED emission, and the hatched region the wavelength interval corresponding to the absorption maxima of the reacted nitrite (NO_2_^–^) reagent. The spectral overlap of these three elements defines an effective interaction window in which light transmitted through the waveguide is strongly absorbed by the dye. This spectral matching ensures efficient coupling between the guided light and the colored reaction products in the photometric chip.

**Figure 3:**
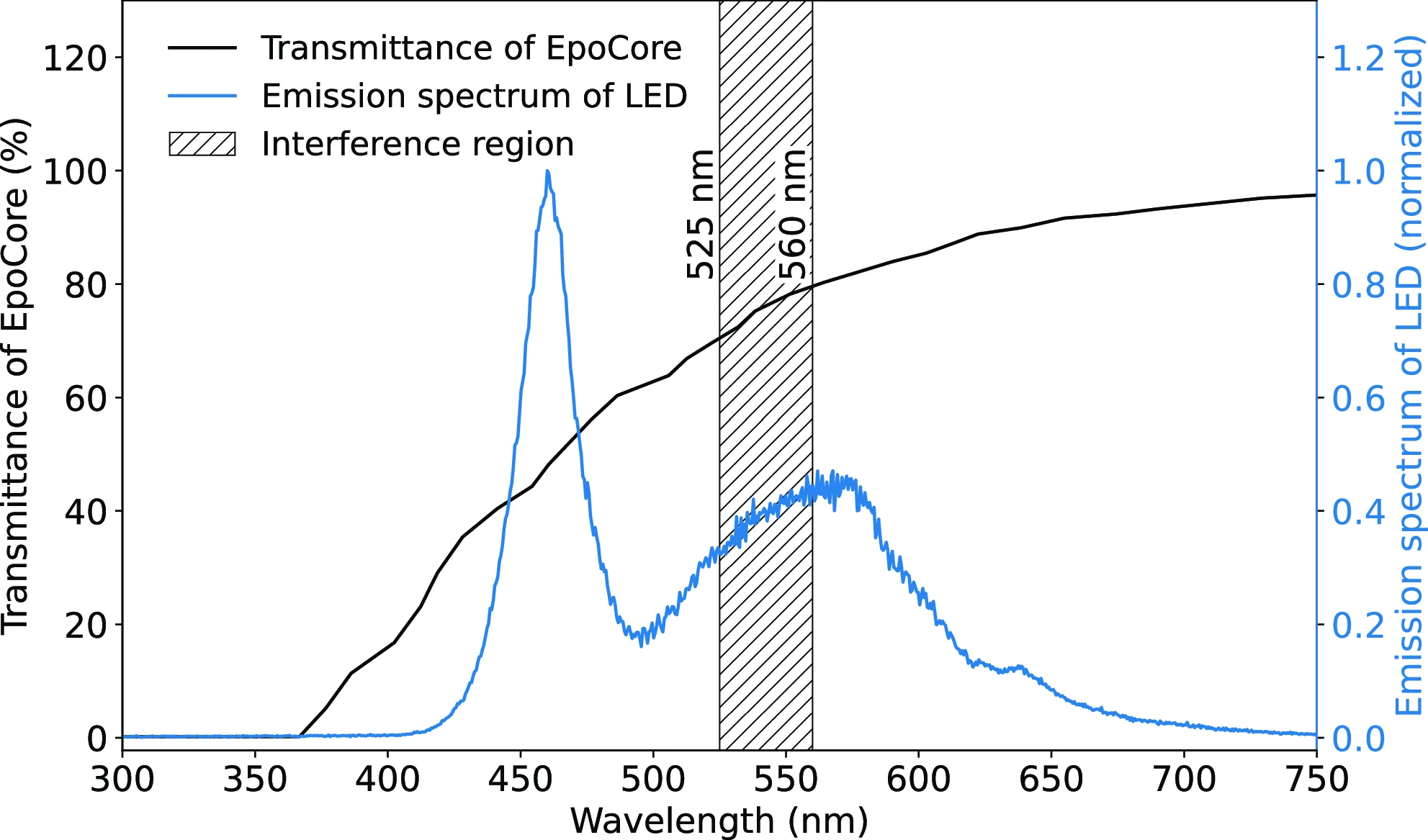
Spectral overlap of the polymer waveguide transmittance (black line), the normalized LED emission spectrum (blue line), and the absorption band of the nitrite test reagent after reaction with nitrite (NO_2_^–^) (hatched region). The hatched region indicates the wavelength range (≈525–560 nm) where waveguide transmission, LED emission, and reagent absorbance coincide, thereby providing efficient interaction between the guided light and the colored reaction products in the photometric chip.

### 3.2 Spectrometric Analysis of Test Solutions

Before measurements with the polymer lab-on-a-chip and smartphone, we evaluated the suitability of the smartphone flash LED as a light source for the NO_2_^–^ test kit by spectrometric analysis. An optical fiber with a 100 μm core and a 3 mm-thick cuvette in the middle was used as a simple waveguide–cell arrangement: one fiber end was coupled to the smartphone LED and the other to a spectrometer (AvaSpec-2048, Avantes). Dye solutions prepared from distilled water samples with different nitrite concentrations were introduced into the cuvette, and the corresponding transmission spectra were recorded (Figure 4a). The spectra show significant absorption over the full measured range, including 500–600 nm, which overlaps the polymer waveguide transmission band and the absorption maxima of the nitrite test reagent. The light intensity at 540 nm decreased systematically with increasing nitrite concentration, and the absorbance–concentration dependence was well described by a four-parameter logistic (4PL) fit with *R* ^2^ = 0.995 (Figure 4b).^41–43^ For subsequent measurements with the CMOS sensor, we used a Beer–Lambert linear calibration model, which is well established for such photometric applications. These results indicate that the concentration-dependent modulation of the optical signal is sufficiently strong to be detected by an image sensor when a polymer waveguide is integrated into the lab-on-a-chip system.

**Figure 4:**
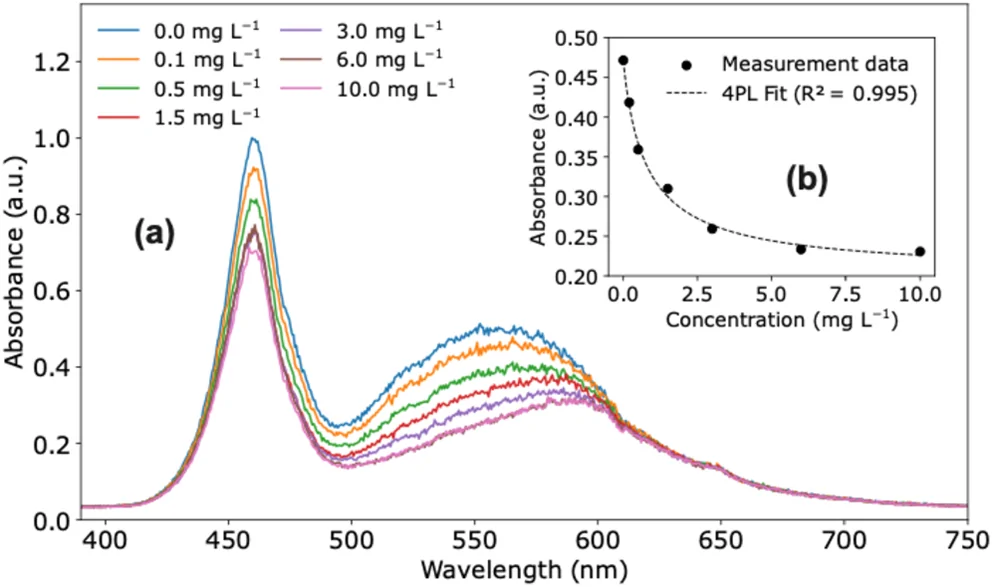
Spectrometric characterization of the NO_2_^–^ test solutions using the smartphone flash LED as a light source. (a) Absorbance spectra of dye solutions prepared from water samples with nitrite concentrations of 0.0, 0.1, 0.5, 1.5, 3.0, 6.0, and 10.0 mg L^–1^. (b) Absorbance at 540 nm as a function of nitrite concentration fitted with a 4PL model (*R* ^2^ = 0.995).

### 3.3 Measurement Setup

Two experimental setups were evaluated for proof-of-concept measurements. The first was fully smartphone-based, employing the smartphone flash LED and camera (Apple iPhone 6) with a 3D-printed adapter and chip cartridge for integration (Figure 5a). In this configuration, only one detection cell was realized, as illustrated in Figure 5b by a representative signal image obtained with this setup. However, using the smartphone camera as a detector poses specific programming challenges, including the need for advanced control of camera focus and limited access to raw Bayer data, which complicate reproducible measurements and calibration of the full system. For a fully smartphone-based implementation, the camera interface must (i) provide stable access to raw Bayer data, (ii) allow fixed and reproducible exposure and gain settings, and (iii) permit internal image processing and automatic adjustments to be disabled. These requirements are not yet consistently met by current commercial smartphones and their application programming interfaces (APIs). We therefore adopted a hybrid configuration to ensure quantitative control in this proof-of-concept study and leave full on-phone integration to future development.

**Figure 5:**
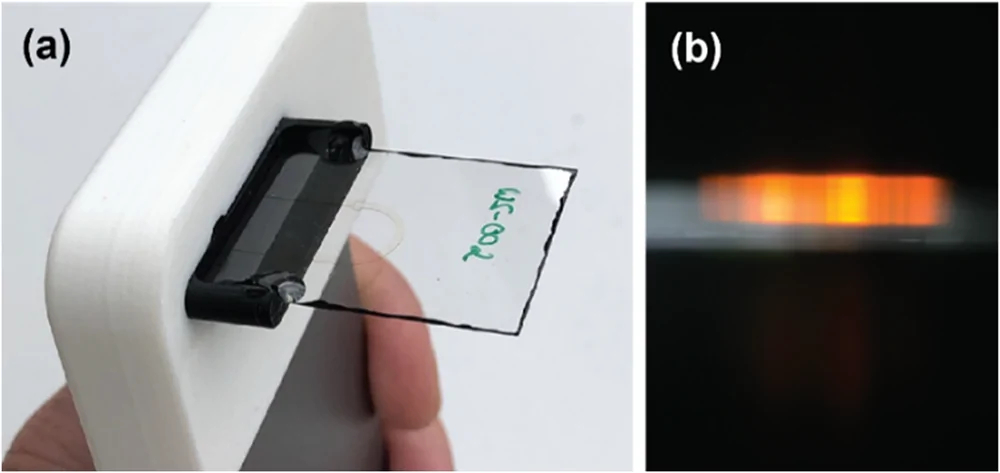
Smartphone-based measurement setup. (a) 3D-printed adapter with polymer optofluidic chip mounted in front of the smartphone flash LED and camera. (b) Example of a signal image recorded with the smartphone camera, showing the intensity distribution of the waveguide outputs.

To enable full control over imaging parameters and facilitate concept validation, we used a development camera board (Cam H7 Plus, OpenMV) in combination with the smartphone flash LED (Figure 6a).

**Figure 6:**
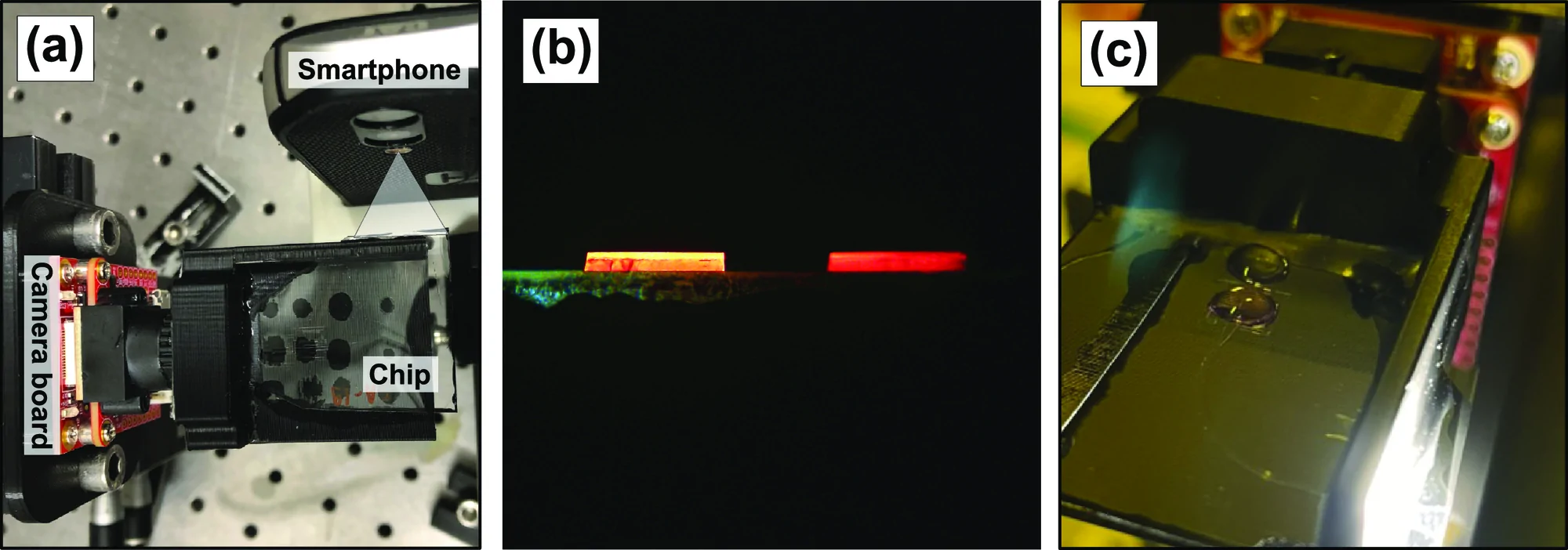
Smartphone-compatible measurement setup using the development camera board and smartphone flash LED. (a) Experimental arrangement with the polymer optofluidic chip mounted in a 3D-printed adapter between the smartphone (flash LED as light source) and the OpenMV Cam H7 Plus camera board. (b) Example of a manually focused signal image showing the two illuminated waveguide outputs corresponding to the measurement and reference channels. (c) 200 μL samples pipetted into the measurement and reference cells, respectively.

In this configuration, the smartphone flash LED serves as the light source, while the development camera board provides precise manual control of exposure and gain for reproducible measurements. Figure 6b shows a manually focused image of the waveguide outputs acquired with this arrangement, and Figure 6c illustrates the two microfluidic detection cells filled with 200 μL of sample in the measurement and reference channels, respectively. This approach preserved the core measurement concept while ensuring reliable and reproducible data acquisition. Thus, the current experiments demonstrate a smartphone-compatible optofluidic module rather than a fully integrated smartphone product, with this configuration chosen to prioritize quantitative validation over limited app-level camera control.

### 3.4 Detector Validation and Signal Image Processing

In practice, the full dynamic range of the CMOS detector cannot be used because linearity deteriorates near saturation and can trigger internal automatic adjustments. The camera board therefore allows the automatic functions, such as auto gain control, auto white balance, auto exposure control, night mode, and backlight compensation (BLC) to be disabled or manually configured. For all nitrite measurements, night mode, BLC, and auto white balance were turned off. The gain was fixed at 1 dB and the exposure time at 0.1 s. These settings were essential to obtain quantitative light-intensity readings as a function of sample concentration.

To validate these settings and the linearity of the CMOS sensor, we illuminated the detector with an adjustable fiber-coupled broadband superluminescent LED (SLED, Exalos; center wavelength 840 nm). The incident radiant power was split by a 3 dB coupler, with one path directed to the CMOS sensor and the other to a calibrated photodiode power sensor (S405C with PM400, Thorlabs) for reference. Figure 7 compares the sensor response in fully automatic mode and in the manually tuned mode (Gain: 36 dB; Exposure time: 0.16 s). In automatic mode, internal camera control causes noticeable nonlinearities, whereas the manually tuned mode exhibits a linear relationship between digital pixel output and optical power and suppresses unwanted internal adjustments, providing approximately 14× higher sensitivity and improved accuracy.

**Figure 7:**
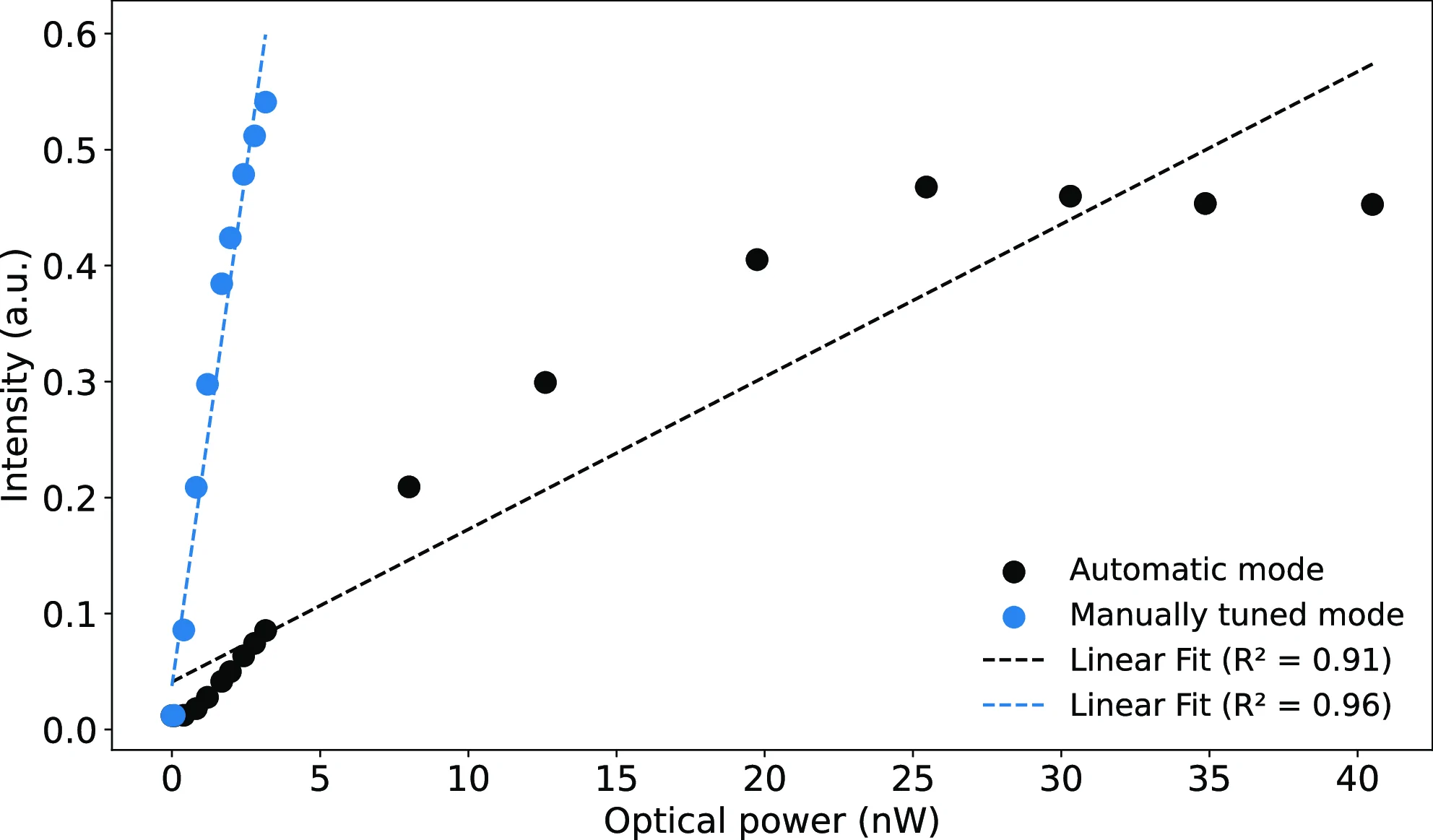
Response of the OV5640 CMOS sensor in fully automatic mode and in manually tuned mode, showing improved linearity and sensitivity in the manually tuned configuration with fixed gain and exposure settings.

After validating the CMOS sensor, we integrated it into the lab-on-a-chip measurement setup. Figure 8a shows a representative camera image (QVGA, 320 × 260 pixels) of the polymer sensor chip, in which both waveguide outputs (test measurement and reference) are visible.

**Figure 8:**
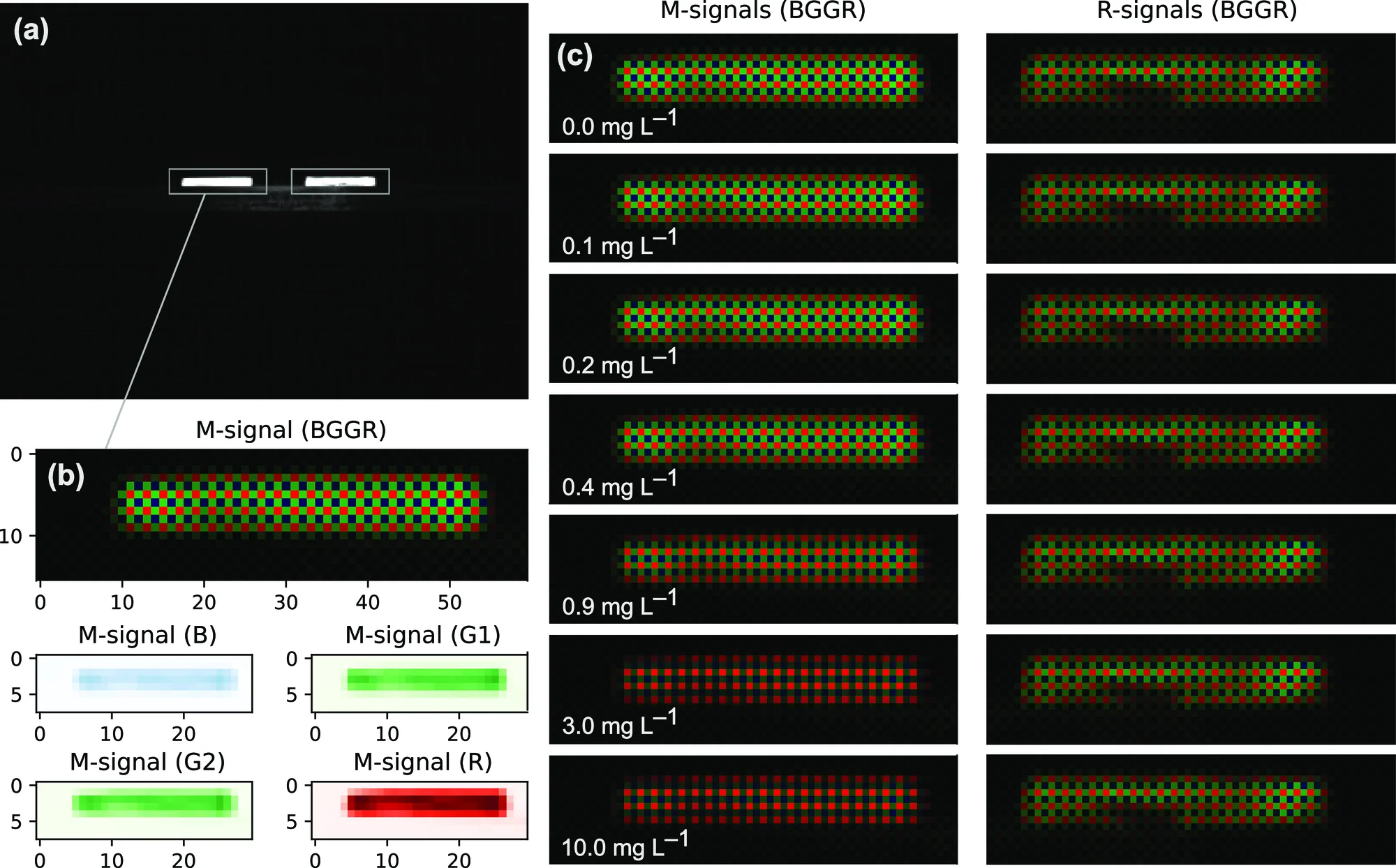
(a) Full camera image obtained with the lab-on-a-chip measurement setup, showing the measurement and reference waveguide outputs of the polymer sensor chip. (b) Selected regions of interest containing the measurement signal, recorded in raw Bayer format for quantitative analysis. (c) Measurement and reference signal intensities for nitrite concentrations between 0.0 and 10.0 mg L^–1^.

For quantitative analysis, image sections containing the measurement and reference waveguide outputs were selected (Figure 8b). These signal images were acquired at a resolution of 16 × 60 pixels in raw Bayer format, providing unprocessed 8-bit RGB pixel intensities without interpolation and enabling channel-specific evaluation of the transmitted light for different nitrite concentrations, as shown in Figure 8c. Within the 0–10 mg L^–1^ range, the T-signal exhibits clear concentration-dependent variations: the green (G1, G2) channels show the largest relative changes, whereas the red channel (R) responds noticeably more weakly.

## 4 Results

### 4.1 Nitrite Detection

Figure 9 shows the RGB-channel responses of the CMOS sensor to reagent-treated water samples with nitrite concentrations in the range 0.0–3.0 mg L^–1^. All three channels exhibit a decrease in signal intensity with increasing NO_2_^–^ concentration, with the green channel providing the highest sensitivity and lowest limit of detection. For the green channel (G), the LoD and LoQ are 0.04 mg L^–1^ and 0.12 mg L^–1^, respectively, calculated as three and ten times the standard deviation of the blank divided by the sensor sensitivity (see eq 4). The shaded region in the green-channel plot indicates the 95% confidence interval (CI) of the linear Beer–Lambert calibration fit. Error bars represent the standard deviations from ten repeated image acquisitions at each nitrite concentration for the same chip loading.

**Figure 9:**
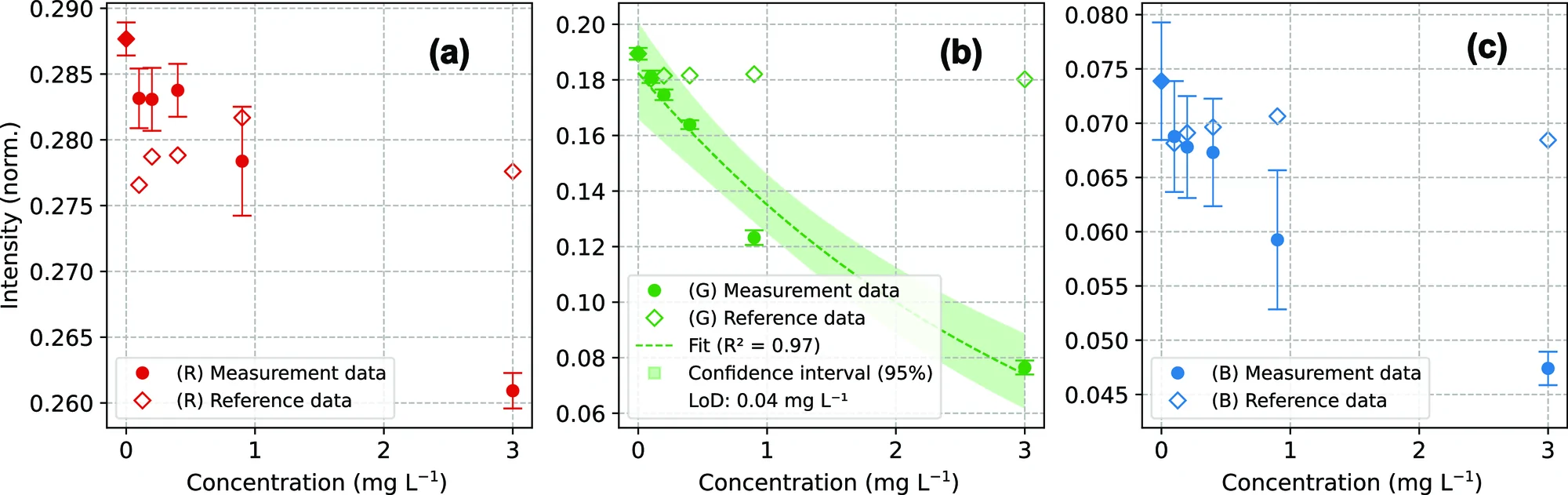
Nitrite sensing performance of the polymer optofluidic chip for the (a) red, (b) green, and (c) blue CMOS channels. Filled circles represent measurement data, and open diamonds represent reference-channel data. For the green (G) channel, the dashed line shows the linear Beer–Lambert calibration fit (*R* ^2^ = 0.97), and the shaded band indicates the corresponding 95% confidence interval; the resulting LoD is 0.04 mg L^–1^. Error bars represent the standard deviations from ten repeated measurements (ten repeated image acquisitions at each concentration).

### 4.2 Chip Performance and Reproducibility

To assess chip-to-chip reproducibility, five independently fabricated chips (Chips 1–5) were tested over the same concentration range. All chips exhibit a decrease in normalized signal intensity with increasing analyte concentration and yielded highly linear calibration curves, with coefficients of determination *R* ^2^ between 0.964 and 0.991, as shown in Figure 10. The LoD and LoQ for the five chips ranged from 0.04 to 0.14 and from 0.12 to 0.48 mg L^–1^, respectively (average: 0.08 and 0.25 mg L^–1^). These results demonstrate excellent linearity and good reproducibility between chips, with only minor variation in LoD values across the five devices, indicating a robust fabrication and measurement procedure under the tested conditions.

**Figure 10:**
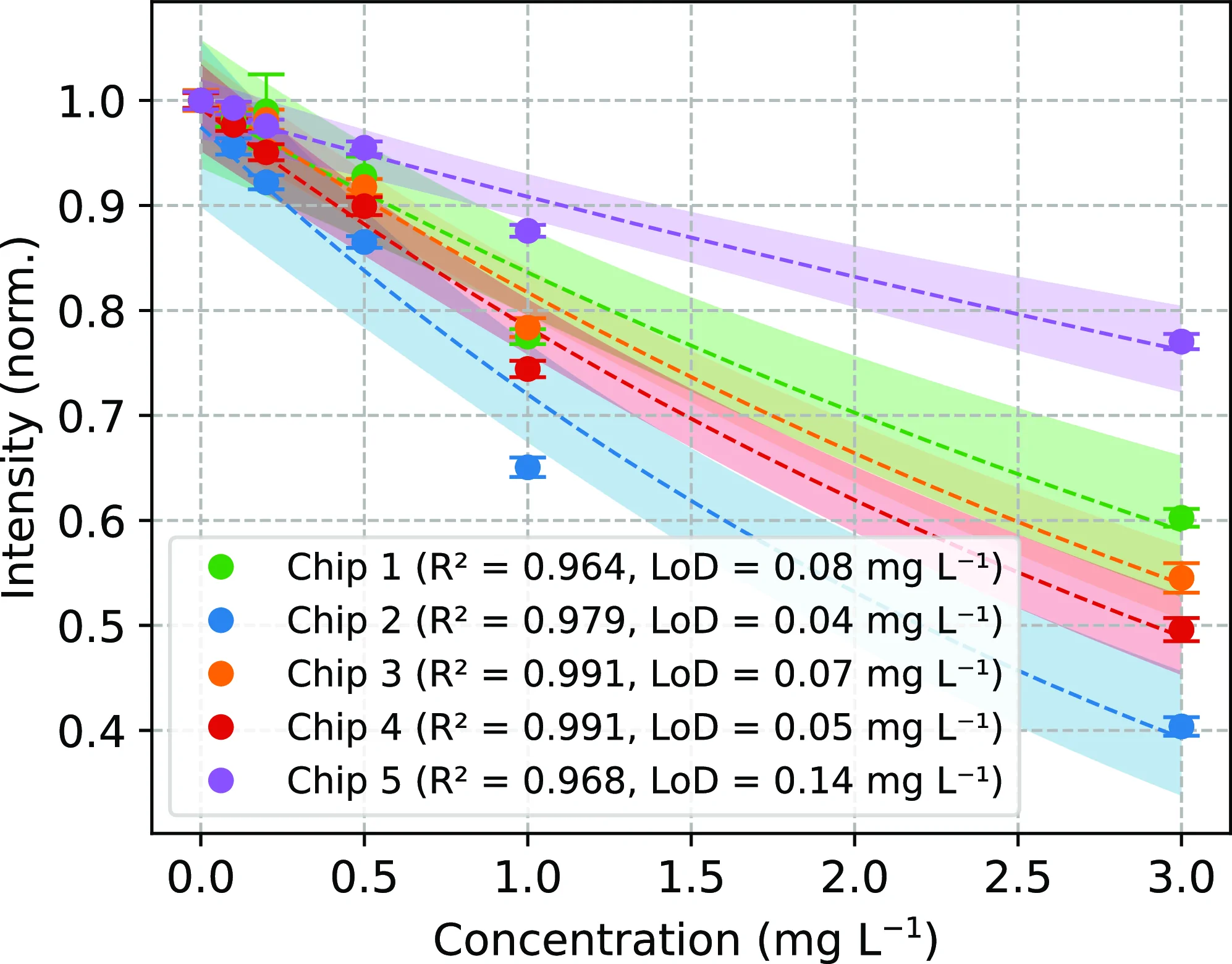
Normalized intensity as a function of analyte concentration for five independently fabricated chips (Chips 1–5). Symbols show the mean normalized intensity at each concentration; error bars denote standard deviations (*n* = 10 repeated image acquisitions at each concentration). Dashed lines correspond to linear Beer–Lambert calibration fits. The coefficients of determination *R* ^2^ and LoD for each chip are indicated in the legend.

### 4.3 Validation with Tap and Surfase Waters

To evaluate the performance of the proposed polymer waveguide chip in representative real-water matrices, we analyzed tap water and surface water samples and quantified nitrite using calibration curves prepared from standard nitrite solutions. Method accuracy in each matrix was assessed by spike–recovery experiments.

For validation in tap water and surface water matrices, two chips were first calibrated using nitrite standard solutions spanning the linear working range of 0.0–1.8 mg L^–1^. Standards at 0, 0.1, 0.2, 0.4, 0.9, and 1.8 mg L^–1^ were measured with *n* = 3 replicate measurements (three repeated fluidic injections) per concentration. The average response at each concentration was then used to fit a Beer–Lambert-type calibration (black scatters and dashed lines in Figure 11). The shaded region indicates the 95% confidence interval (CI) of the linear Beer–Lambert calibration fit.

**Figure 11:**
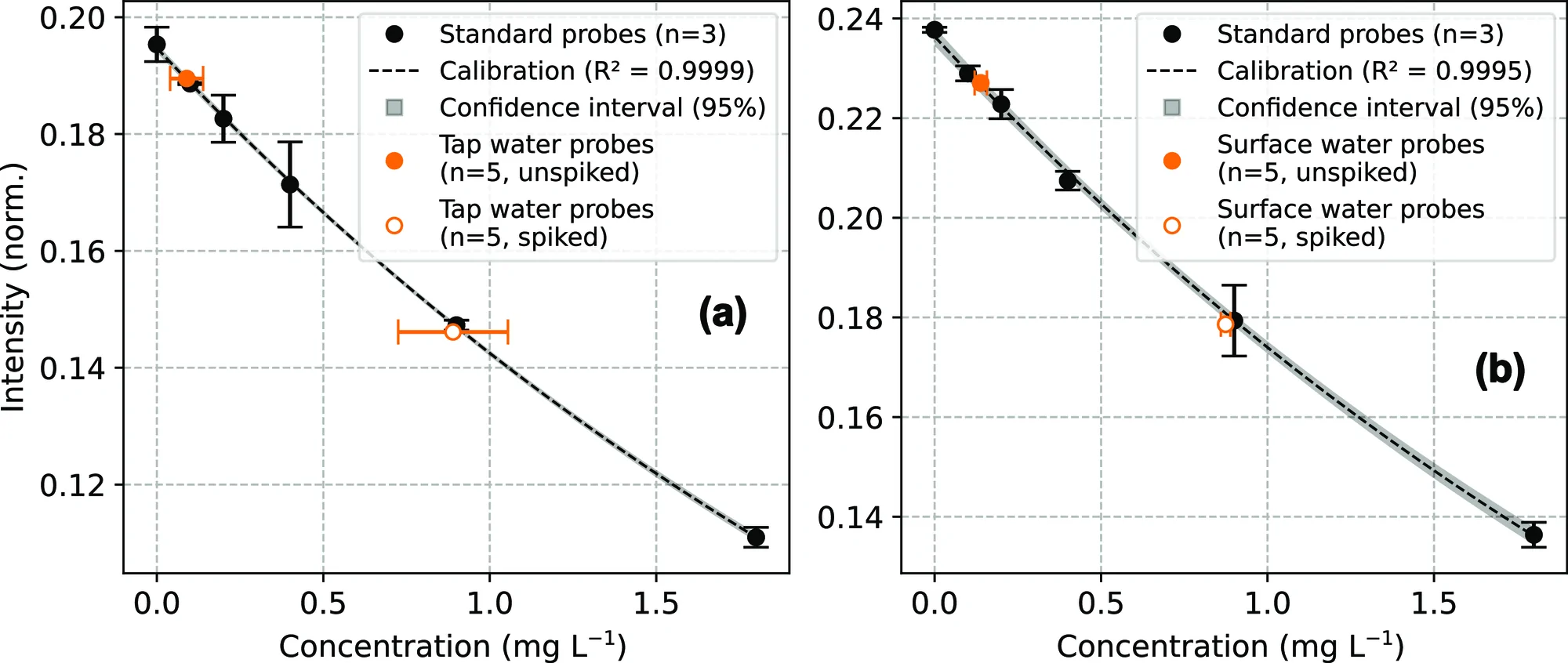
Chip calibration using nitrite standard solutions in the range 0.0–1.8 mg L^–1^ (black scatters and dashed line: Beer–Lambert linear fit) and corresponding measurements of unspiked (solid filled orange scatters) and spiked samples (unfilled orange scatters): (a) for tap water and (b) for surface water matrices.

After calibration, tap water and surface water samples were collected as *n* = 5 independent samples (Goslar, Germany, 5–7 June 2026) and measured as received, with five independent sample preparations. Based on the measured intensities and the calibration curve, the nitrite concentration in the unspiked tap water was 0.09 ± 0.05 mg L^–1^ (mean ± SD, *n* = 5), while the unspiked surface water contained 0.14 ± 0.02 mg L^–1^ (solid filled orange scatters in Figure 11a,b).

To assess matrix effects, the same samples were subsequently spiked with nitrite at +0.9 mg L^–1^. The measured concentrations after spiking were 0.89 ± 0.16 mg L^–1^ for tap water and 0.87 ± 0.01 mg L^–1^ for surface water (unfilled orange scatters in Figure 11a,b). Persent spike recovery was calculated according to ref 44 as

5 $$\%\mathrm{recovery}=\frac{X_{s}-X_{u}}{K}\times 100\%$$

where *X*_s_ is measured concentration in the spiked sample, *X*_u_ is the measured concentrationin in the corresponding unspiked sample, and *K* is the known added nitrite concentration in the spike. Using this definition, the mean recoveries were 89 ± 15% for the tap water samples and 82 ± 2% for the surface water samples (Table 1).

**Table 1:** Spike–Recovery Results for Nitrite in Tap-Water and Surface-Water Sampels

	tap-water validation			surface-water validation		
sample number	unspiked (mg L^–1^)	spiked (mg L^–1^)	recovery (%)	unspiked (mg L^–1^)	spiked (mg L^–1^)	recovery (%)
1	0.13	0.79	73	0.16	0.89	81
2	0.15	1.18	114	0.12	0.87	83
3	0.07	0.89	91	0.16	0.88	80
4	0.05	0.80	84	0.12	0.85	81
5	0.05	0.80	84	0.13	0.88	84
mean ± SD (RSD%)	0.09 ± 0.05 (55.6%)	0.89 ± 0.16 (18%)	89 ± 15 (17%)	0.14 ± 0.02 (14%)	0.87 ± 0.01 (12%)	82 ± 2 (2%)

## 5 Discussion

The results demonstrate that maskless laser-direct UV lithography enables reproducible fabrication of polymer-based optofluidic lab-on-a-chip devices, including complex layouts with integrated couplers. This approach supports on-chip visible photometry with functionality comparable to conventional double-beam systems. The integrated reference cell compensates for fluctuations of the smartphone flash LED, thereby improving signal stability and reliability.

The average chip-to-chip LoD of the green channel is 0.08 mg L^–1^, clearly below the European Union drinking-water limit of 0.5 mg L^–1^. From the same calibration data, the corresponding LoQ is approximately 0.25 mg L^–1^.

### 5.1 Benchmarking

Table 2 benchmarks the proposed system against representative commercial analyzers and selected recently reported sensors. For each device, the table lists the analyte, sensing principle, measuring range, limit of detection (LoD), sample volume and reagent consumption, and response time (excluding handling).

**Table 2:** Comparison of the Proposed Optofluidic Sensor with Selected Commercial and Literature-Reported Nitrite Sensors^a^

device (company)	analyte	principle	measuring range (mg L^–1^)	LoD (mg L^–1^)	sample volume/reagent consumption	reaction time (min)	refs
Commercial Approaches							
DR3900 + LCK342 reagent (Hach)	NO_2_^–^	colorimetric	0.6–6	NR	0.2 mL/NR	10	^45,46^
test Kit 1.14774 (Merck)	NO_2_^–^	colorimetric	0.1–10	NR	6.0 mL/0.08 g	3	^47^
Scientifically Reported Approaches							
dual-mode nitrite sensor	NO_2_^–^	colorimetric	0–20	0.05	0.6 mL/NR	7	^48^
		fluorometric		0.6			
paper-based nanosensor	NO_2_^–^	CQDs on PAD	1–100 ppm	0.1 ppm	2.0 mL/100 μL of 100 mM HCl	30	^12^
paper-based sensor	NO_2_^–^	colorimetric	0.5–40	0.1	20 μL/4 μL	5	^49^
polymer optofluidic chip	NO_2_^–^	photometric	0.1–3	0.08	1.0 mL/0.01 g	3	this work

a NR—not reported.

For the proposed platform, the reaction (color-development) time is 3 min, and including sample loading and image acquisition the time-to-result is on the order of a few minutes. Each test uses a 1 mL prepared sample, whereas the chip itself requires only 200 μL per channel (measurement and reference, see Figure 6c), corresponding to 400 μL total volume loaded per measurement. The color-reagent consumption is approximately 0.01 g per 1 mL of sample. The approximate hardware cost of the optical readout (CMOS sensor, LED, and associated electronics) is below 50 USD. In our current lab-scale process using a rapid-prototyping maskless UV-lithography tool, a single lithography run yields four chips in a few hours with a functional yield above 90%. For industrial-scale fabrication, conventional mask UV lithography or replication techniques (e.g., hot embossing) could be employed for mass production and are expected to reduce the chip cost to well below 1 USD per piece, depending on volume and process optimization. The reagents needed for one nitrite assay are approximately 0.1 USD. Operational complexity is moderate and involves only a few user steps: prepare the reagent, load the sample into both channels, wait 3 min, and capture and process an image.

Commercial benchtop spectrophotometers offer well-established laboratory performance but require relatively expensive instrumentation and cuvette-based tests, and are not inherently portable. Portable photometers improve field deployability but still rely on dedicated hardware and proprietary consumables. In this context, the proposed system complements recent hand-held portable devices and smartphone-integrated platforms for nitrite monitoring by providing a reusable, low-cost polymer chip operated with widely available commercial reagents and read out by a compact CMOS image sensor. The optofluidic chip itself is a passive component that can be mass produced at low cost, while the readout relies either on a small external module incorporating a low-cost camera and LED or on a smartphone-compatible platform, resulting in a portable setup suitable for hand-held operation. Accordingly, the comparison in Table 2 indicates that the proposed system is cost-effective and portable, while still offering analytically relevant limits of detection and measurement ranges for decentralized nitrite monitoring.

### 5.2 Limitations and Future Work

The present work demonstrates the proof-of-concept and analytical performance of the polymer waveguide platform under controlled laboratory conditions and in two representative real-water matrices (tap water and surface water). Calibration with nitrite standards in the range 0.0–1.8 mg L^–1^ and spike–recovery experiments yielded mean recoveries of 89 ± 15% for tap water and 82 ± 2% for surface water, indicating acceptable accuracy in these matrices. Nevertheless, the current validation is limited to clear, relatively low-turbidity waters from a single geographical location. Systematic matrix studies, combined with benchmarking against accredited reference methods, will be necessary to fully quantify matrix effects, recovery, accuracy, and robustness under field-relevant conditions.

Chip-to-chip reproducibility and repeated-measurement statistics were evaluated, but long-term operational stability, environmental robustness, and aging of the polymer waveguide system were not addressed. In practical field deployment, temperature and humidity variations, mechanical handling, prolonged optical exposure, and material aging may affect baseline stability and sensitivity. Dedicated stability and stress-testing campaigns under realistic operating and storage conditions therefore remain an important task for future work.

From a manufacturing perspective, large-scale production will require tight control of the waveguide cleaving process to obtain clean, reproducible input and output facets, thereby reducing the need for individual chip calibration and ensuring consistent optical performance across batches.

Finally, while the two-channel configuration implements the double-beam principle on chip, it is not analytically equivalent to a classical double-beam spectrophotometer with a dedicated reference detector monitoring the incident intensity. In the present architecture, both signals propagate through separate fluidic channels; residual channel-to-channel differences in waveguide loss, as well as differences in wetting, filling, and fouling behavior, may introduce systematic offsets and gradually degrade referencing quality during long-term operation. Future developments will therefore focus on refining the referencing scheme, quantifying its long-term stability, and integrating these insights into sensor design and calibration strategies for field deployment.

## 6 Conclusion

In summary, we have demonstrated a compact, cost-effective polymer optofluidic lab-on-a-chip, fabricated by maskless laser-direct UV lithography, for double-beam photometric determination of nitrite in water using standard RGB CMOS image sensors. The device integrates polymer optical waveguides, an on-chip 3 dB coupler, and dual microfluidic cells to realize a measurement/reference configuration operated with commercial Griess-reaction-based colorimetric test kits. For reagent-treated standard water samples, limits of detection of 0.08 mg L^–1^ were achieved, which are well suited for typical drinking-water and environmental monitoring scenarios defined by the European Drinking Water Directive, provided that matrix effects in real samples are appropriately controlled or calibrated. The chip operates passively, using the smartphone flash LED as a broadband visible light source and the CMOS sensor as detector, thereby eliminating the need for benchtop spectrophotometers and enabling a portable hand-held or smartphone-compatible measurement concept for nitrite monitoring.

As a next development step, the platform can be extended toward multiwaveguide, multianalyte configurations with on-chip baseline referencing, using a single RGB CMOS image sensor to monitor multiple signals with one broadband light source (see Figure 8). Future work will focus on refining waveguide cleaving, quantifying coupling efficiency and effective optical path length, evaluating alternative polymer materials and reagent kits to further improve robustness and sensitivity, and extending the platform to additional colorimetric assays for multiparameter water-quality analysis. Owing to its low-cost fabrication, scalable design, and compatibility with hand-held devices, the presented optofluidic chip represents a promising analytical platform for dense, decentralized monitoring of nitrite in drinking-water distribution networks, wastewater treatment and reuse facilities, and agricultural settings. By systematically addressing the limitations identified in this study, we aim to advance the current proof-of-concept toward a more robust, prototype-level device suitable for routine field use.

## References

[1] Drewes J. E., Khan S. J. (2015). Contemporary Design, Operation, and Monitoring of Potable
Reuse Systems. J. Water Reuse Desalin..

[2] Morseletto P., Mooren C. E., Munaretto S. (2022). Circular Economy of Water: Definition,
Strategies and Challenges. Circ. Econ. Sustainability.

[3] Yang K., Peretz-Soroka H., Liu Y., Lin F. (2016). Novel Developments
in Mobile Sensing Based on the Integration of Microfluidic Devices
and Smartphones. Lab. Chip.

[4] Li Z., Liu H., Wang D., Zhang M., Yang Y., Ren T. (2023). Recent Advances
in Microfluidic Sensors for Nutrients Detection in Water. TrAC Trends Anal. Chem..

[5] Guidelines for Drinking-water Quality: Fourth Edition Incorporating The First And Second Addenda. https://www.who.int/publications/i/item/9789240045064. (accessed January 5, 2026). 35417116

[6] Nitrate and Nitrite in Drinking-Water; World Health Organization. https://www.who.int.

[7] Guo J., Liu M. J., Laguna C., Miller D. M., Williams K. S., Clark B. D., Muñoz C., Blair S. J., Nielander A. C., Jaramillo T. F., Tarpeh W. A. (2024). Electrodialysis and Nitrate Reduction
(EDNR) to Enable Distributed Ammonia Manufacturing from Wastewaters. Energy Environ. Sci..

[8] European Water Policies and Human Health—Combining Reported Environmental Information; EEA. https://www.eea.europa.eu/en/analysis/publications/public-health-and-environmental-protection. (accessed January 5, 2026).

[9] Biswas P. C., Rani S., Hossain M. A., Islam M. R., Canning J. (2021). Recent Developments
in Smartphone Spectrometer Sample Analysis. IEEE J. Sel. Top. Quantum Electron..

[10] Di
Nonno S., Ulber R. (2021). Smartphone-Based Optical Analysis
Systems. Analyst.

[11] Siu V. S., Lu M., Hsieh K. Y., Raines K., Asaad Y. A., Patel K., Afzali-Ardakani A., Wen B., Budd R. (2022). Toward a Quantitative
Colorimeter for Point-of-Care Nitrite Detection. ACS Omega.

[12] Oroujlo M., Bagheri Z., Naghdi T., Golmohammadi H. (2025). Smart Paper-Based
Nanosensor for Simultaneous Environmental eMonitoring of Nitrate and
Nitrite. ACS Meas. Sci. Au.

[13] Xu K., Chen Q., Zhao Y., Ge C., Lin S., Liao J. (2020). Cost-Effective, Wireless, and Portable
Smartphone-Based Electrochemical
System for on-Site Monitoring and Spatial Mapping of the Nitrite Contamination
in Water. Sens. Actuators, B.

[14] Autonomous Sensor Networks: Collective Sensing Strategies for Analytical Purposes; Filippini, D. , Ed.; Springer Series on Chemical Sensors and Biosensors; Springer Berlin Heidelberg: Berlin, Heidelberg, 2013; Vol. 13.

[15] Ali, Md. A. ; Jiao, Y. ; Tabassum, S. ; Wang, Y. ; Jiang, H. ; Dong, L. Electrochemical Detection of Nitrate Ions in Soil Water Using Graphene Foam Modified by TiO2 Nanofibers and Enzyme Molecules; 2017 19th International Conference on Solid-State Sensors, Actuators and Microsystems (TRANSDUCERS); IEEE: Kaohsiung, 2017; pp 238–241 10.1109/TRANSDUCERS.2017.7994032.

[16] Jaywant S. A., Arif K. M. (2019). A Comprehensive
Review of Microfluidic Water Quality
Monitoring Sensors. Sensors.

[17] Jayawardane B. M., Wei S., McKelvie I. D., Kolev S. D. (2014). Microfluidic Paper-Based Analytical
Device for the Determination of Nitrite and Nitrate. Anal. Chem..

[18] Ferreira F. T. S. M., Mesquita R. B. R., Rangel A. O. S. S. (2020). Novel
Microfluidic
Paper-Based Analytical Devices (μPADs) for the Determination
of Nitrate and Nitrite in Human Saliva. Talanta.

[19] Morgan R. L., Zehnder L. J., Jenkin M. A., Alonso E., Haunz S., Bevil A., Scott D. F. (2025). Engineered
Nanoparticles for μPAD
Nucleic Acid Detection. ACS Omega.

[20] Lopez-Ruiz N., Curto V. F., Erenas M. M., Benito-Lopez F., Diamond D., Palma A. J., Capitan-Vallvey L. F. (2014). Smartphone-Based
Simultaneous pH and Nitrite Colorimetric Determination for Paper Microfluidic
Devices. Anal. Chem..

[21] Fàbrega C., Fernández L., Monereo O., Pons-Balagué A., Xuriguera E., Casals O., Waag A., Prades J. D. (2017). Highly
Specific and Wide Range NO_2_ Sensor with Color Readout. ACS Sens..

[22] Ortiz-Gomez I., Ortega-Muñoz M., Salinas-Castillo A., Álvarez-Bermejo J. A., Ariza-Avidad M., De Orbe-Payá I., Santoyo-Gonzalez F., Capitan-Vallvey L. F. (2016). Tetrazine-Based
Chemistry for Nitrite Determination
in a Paper Microfluidic Device. Talanta.

[23] Beaton A. D., Cardwell C. L., Thomas R. S., Sieben V. J., Legiret F.-E., Waugh E. M., Statham P. J., Mowlem M. C., Morgan H. (2012). Lab-on-Chip
Measurement of Nitrate and Nitrite for In Situ Analysis of Natural
Waters. Environ. Sci. Technol..

[24] Adarsh N., Shanmugasundaram M., Ramaiah D. (2013). Efficient Reaction Based Colorimetric
Probe for Sensitive Detection, Quantification, and On-Site Analysis
of Nitrite Ions in Natural Water Resources. Anal. Chem..

[25] Czugala M., Fay C., O’Connor N. E., Corcoran B., Benito-Lopez F., Diamond D. (2013). Portable Integrated Microfluidic Analytical Platform
for the Monitoring and Detection of Nitrite. Talanta.

[26] Khanfar M., Al-Faqheri W., Al-Halhouli A. (2017). Low Cost Lab on Chip for the Colorimetric
Detection of Nitrate in Mineral Water Products. Sensors.

[27] Luka G. S., Nowak E., Kawchuk J., Hoorfar M., Najjaran H. (2017). Portable Device
for the Detection of Colorimetric Assays. R.
Soc. Open Sci..

[28] Dudala S., Dubey S. K., Goel S. (2019). Fully Integrated, Automated, and
Smartphone Enabled Point-of-Source Portable Platform With Microfluidic
Device for Nitrite Detection. IEEE Trans. Biomed.
Circuits Syst..

[29] Gu Z., Wu M.-L., Yan B.-Y., Wang H.-F., Kong C. (2020). Integrated
Digital Microfluidic Platform for Colorimetric Sensing of Nitrite. ACS Omega.

[30] Wang F., Zhu J., Hu X., Chen L., Zuo Y., Yang Y., Jiang F., Sun C., Zhao W., Han X. (2021). Rapid Nitrate
Determination with a Portable Lab-on-Chip Device Based on Double Microstructured
Assisted Reactors. Lab. Chip.

[31] Harris, D. C. ; Lucy, C. A. Quantitative Chemical Analysis; W. H. Freeman: Austin Boston New York Plymouth, 2019.

[32] Qi Y., Zheng Z., Banakar M., Wu Y., Gangnaik A., Rowe D. J., Mittal V., Butement J., Wilkinson J. S., Mashanovich G. Z., Nedeljkovic M. (2021). Integrated
Switching Circuit for
Low-Noise Self-Referenced Mid-Infrared Absorption Sensing Using Silicon
Waveguides. IEEE Photonics J..

[33] Bethmann, K. ; Orghici, R. ; Pichler, E. ; Zywietz, U. ; Schimdt, T. ; Gleissner, U. ; Kelb, C. ; Roth, B. ; Reinhardt, C. ; Willer, U. ; Schade, W. New Design for a Wavelength Demultiplexing Device; Pickrell, G. ; Udd, E. ; Du, H. H. , Eds.; Proc. SPIE 9480, Fiber Optic Sensors and Applications XII; SPIE: Baltimore, Maryland, United States, 2015. 948010. 10.1117/12.2183328.

[34] Nitrat Küvetten-Test. https://de.hach.com/nitrat-kuvetten-test-0-23-13-5-mg-l-no-n-25-bestimmungen/. (accessed April 14, 2026).

[35] Nitrite Test Kit, colorimetric 0.1–10 mg/L (NO2−), MQuant. https://www.sigmaaldrich.com/DE/en/product/mm/114774#product-documentation. (accessed May 3, 2026).

[36] Color CMOS QSXGA (5 Megapixel) Image Sensor with OmniBSI Technology; OmniVision Technologies. https://cdn.sparkfun.com/datasheets/Sensors/LightImaging/OV5640_datasheet.pdf.

[37] Hussain I., Ahamad K. U., Nath P. (2017). Low-Cost,
Robust, and Field Portable
Smartphone Platform Photometric Sensor for Fluoride Level Detection
in Drinking Water. Anal. Chem..

[38] Oshina I., Spigulis J. (2021). Beer–Lambert
Law for Optical Tissue Diagnostics:
Current State of the Art and the Main Limitations. J. Biomed. Opt..

[39] Little T. A. (2015). Method
Validation Essentials, Limit of Blank, Limit of Detection, and Limit
of Quantitation: Care Needs to Be Made to Match the Method of Limit
Determination to the Analytical Method. Biopharm
Int..

[40] Váradi L., Breedon M., Chen F. F., Trinchi A., Cole I. S., Wei G. (2019). Evaluation of Novel Griess-Reagent Candidates for Nitrite Sensing
in Aqueous Media Identified *via* Molecular Fingerprint
Searching. RSC Adv..

[41] Holstein C. A., Griffin M., Hong J., Sampson P. D. (2015). Statistical Method
for Determining and Comparing Limits of Detection of Bioassays. Anal. Chem..

[42] Dudley R. A., Edwards P., Ekins R. P., Finney D. J., McKenzie I. G., Raab G. M., Rodbard D., Rodgers R. P. (1985). Guidelines for Immunoassay
Data Processing. Clin. Chem..

[43] Busa L. S. A., Maeki M., Ishida A., Tani H., Tokeshi M. (2016). Simple and
Sensitive Colorimetric Assay System for Horseradish Peroxidase Using
Microfluidic Paper-Based Devices. Sens. Actuators,
B.

[44] Patnaik, P. Handbook of Environmental Analysis: Chemical Pollutants in Air, Water, Soil, and Solid Wastes, 3rd ed.; Taylor & Francis Group, CRC Press: Boca Raton, 2018.

[45] DR3900 Spektralphotometer. https://de.hach.com/dr3900-spektralphotometer-mit-rfid-technologie/. (accessed April 14, 2026).

[46] Nitrit Küvetten-Test. https://de.hach.com/nitrit-kuvetten-test-0-6-6-0-mg-l-no-n-25-bestimmungen/. (accessed April 14, 2026).

[47] Nitrite Test Kit 1.14774. https://www.sigmaaldrich.com/DE/de/product/mm/114774. (accessed April 14, 2026).

[48] Liu Y., Qiao W., Fan X., Wang L., Wang M., Liu X., Jia Y., Liu D., Liu T., Zang M., Tian S., Yin F. (2024). Rapid Detection
of Nitrite in Food
by Multi-Colorimetric and Fluorescence Dual-Mode Based on Crystal
Violet Modulated Carbon Dots. Microchem. J..

[49] Thongkam T., Hemavibool K. (2020). An Environmentally
Friendly Microfluidic Paper-Based
Analytical Device for Simultaneous Colorimetric Detection of Nitrite
and Nitrate in Food Products. Microchem. J..

